# Communication about uncertainty and hope: A randomized controlled trial assessing the efficacy of a communication skills training program for physicians caring for cancer patients

**DOI:** 10.1186/s12885-017-3437-8

**Published:** 2017-07-10

**Authors:** Yves Libert, Livia Peternelj, Isabelle Bragard, Aurore Liénard, Isabelle Merckaert, Christine Reynaert, Darius Razavi

**Affiliations:** 1 Université Libre de Bruxelles, Faculté des Sciences Psychologiques et de l’Éducation, Av. F. Roosevelt, 50 (CP 191), 1050 Brussels, Belgium; 2 Institut Jules Bordet, Université Libre de Bruxelles, Brussels, Belgium; 3 Université de Liège, Faculté des Sciences Psychologiques et de l’Éducation, Liège, Belgium; 4 Université Catholique de Louvain, Faculté de Médecine, Brussels, Belgium

**Keywords:** Uncertainty, Hope, Cancer, Communication skills training, Physicians

## Abstract

**Background:**

Although previous studies have reported the efficacy of communication skills training (CST) programs, specific training addressing communication about uncertainty and hope in oncology has not yet been studied. This paper describes the study protocol of a randomized controlled trial assessing the efficacy of a CST program aimed at improving physician ability to communicate about uncertainty and hope in encounters with cancer patients.

**Methods/design:**

Physician participants will be randomly assigned in groups (*n* = 3/group) to a 30-h CST program (experimental group) or to a waiting list (control group). The training program will include learner-centered, skills-focused, practice-oriented techniques. Training efficacy is assessed in the context of an encounter with a simulated advanced stage cancer patient at baseline and after the CST for the experimental group, and after four months for the waiting-list group. Efficacy assessments will include communicational, psychological and physiological measures. Group-by-time effects will be analyzed using a generalized estimating equation (GEE). A power analysis indicated that a sample size of 60 (30 experimental and 30 control) participants will be sufficient to detect effects.

**Discussion:**

The current study will aid in the development of effective CST programs to improve physician ability to communicate about uncertainty and hope in encounters with cancer patients.

**Trial registration:**

US Clinical Trials Register NCT02836197.

## Background

Communication with cancer patients poses a variety of widely recognized challenges for physicians. Breaking bad news and explanations of complex treatments must often be relayed so that decisions can be made. It is therefore important for physicians to provide emotional support to patients and their relatives coping with a disease associated with negative outcomes such as treatment side-effects and shortened life-expectancy. It is important to underline that current cancer treatments are increasingly personalized and based on multidisciplinary approaches [1–3]. Due to medical progress, cancer patients are living longer with their disease, posing new challenges in doctor-patient communication to help patients cope with uncertainty and to promote hope.

Prior studies have indicated that cancer patients have an expectation that physicians will discuss uncertainty and hope [4] to help them adjust to their diagnosis [1, 5, 6] and to maintain hopefulness [7–9]. Moreover, patients wish for realistic, individualized, full and honest information regarding their current medical condition and prognosis [8, 10–14]. These studies suggest that physicians who communicate about uncertainty and hope are meeting patient expectations.

The current models of communication in healthcare advocate the use of general uncertainty management skills during physician communication with cancer patients [15, 16] and assumes that physicians must promote patient hope [17]. Models of coping with cancer [5] argue that communication between physicians and patients about uncertainty may improve patient adjustment to their illness. According to these models, communication about uncertainty and hope will benefit patient quality of life, help the patient maintain a positive outlook and will decrease conflict between physicians and patients when making decisions [1, 17, 18]. These models underline the importance of effective communication skills between physicians and their cancer patients concerning uncertainty and hope [17, 19].

However, due to a lack of specific medical training, physicians often report a negative perception about the outcome of discussing uncertainty and hope with cancer patients [20]. Physicians fear these discussions will lead to unrealistic expectations leading to additional stress on the patient’s condition [20]. In addition, physicians are leery of increasing patient concerns that will be difficult to manage. As a consequence, physicians experience difficulties and can be reluctant to communicate about uncertainty and hope with cancer patients [21–24], fearing that addressing these issues will lead to increased work stress [25].

Poor communication about uncertainty and hope in encounters with cancer patients may lead to negative outcomes for both patients and care providers [25–27]. For cancer patients, poor communication may be detrimental to illness adjustment [28] and may lead to inadequate strategies such as searching certainty, resulting in conflicts with healthcare professionals [29]. For healthcare providers, poor communication may result in a lack of work satisfaction [30], higher risk of burnout [25], higher use of healthcare services [31], increased costs, [25] and decreased quality of care delivery [32]. Studies assessing training methods that may help physicians overcome communication difficulties about uncertainty and hope is thus needed.

Previous studies have reported on the efficacy of communication skills training (CST) programs in the improvement of low- to middle-level communication skills of physicians such as breaking bad news to cancer patients, assessing psychosocial issues and talking with patient relatives [33]. CST programs have used learner-centered, skill-focused and practice-oriented techniques resulting in improvements in physician communication and support skills [34–37], attitudes toward psychosocial and emotional issues [30, 38–41], empathy toward patients [38, 39, 42] and work satisfaction [36]. In addition, these programs have benefited patients by decreasing anxiety [43] and increasing satisfaction [44, 45]. Taken together, the results from these studies have confirmed the usefulness of CST programs offered in small groups (maximum of six participants) over the course of a minimum of 20 h.

The efficacy of CST programs aimed at improving physician communication skills on the topics of uncertainty and hope has not yet been studied [1, 46]. These topics require specific CST. Communicating with patients about uncertainty implies a deep assessment of patient expectations about the future and informing patients about uncertainties. Communication of hope requires a deep understanding of patient wishes for the future while supporting ways needed to achieve them [47]. This collaborative and bidirectional process of communication between physician and patient on sensitive topics associated with the patient’s medical, psychological and social future will ultimately benefit both patient and physician.

## Methods/design

### Aim of the trial

A randomized longitudinal study assessing the efficacy of a CST program aimed at improving physician communication about uncertainty and hope with cancer patients will be conducted. Efficacy of the program will be assessed by the analysis of changes over time in physician communication skills and physician psychological and physiological health. These assessments will be performed in the context of an encounter with a simulated advanced-stage cancer patient.

### Subjects

Participants will be physicians that are specialists or residents, have a practice including cancer patients and speak French. The study was approved by a central ethics committee (Jules Bordet Institute, Cancer Center of the Université Libre de Bruxelles) and all participants will provide written informed consent.

### Study design

**Fig. 1:**
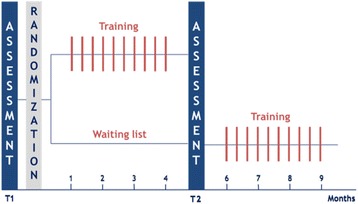
Study Design. Physicians will be randomly assigned to 30-h CST program (*experimental group*) or to a waiting list (*control group*). Training efficacy is assessed in the context of an encounter with a simulated advanced stage cancer patient at baseline and after the CST for the experimental group, and after four months for the waiting list group. Communicational, psychological and physiological assessments will be conducted

Participating physicians will be randomly assigned to either the experimental group or the control group (Fig. 1). After baseline assessment, participants in the experimental group will attend a 4-month training program followed by a post assessment. Participants in the control group will be placed on a waiting list after baseline assessment and will be reassessed four months later. The process of randomization after baseline will allow for a double-blind assessment at baseline.

### CST program

#### CST aims

The aim of the CST is to improve the ability of physicians to communicate about uncertainty and hope with cancer patients.

#### CST logistics

The CST is a manualized program comprised of ten 3-h sessions (30 h) spread over four to five months. Each training group will include three physicians. The training will be conducted at locations and times choosen by the physicians within each group. The trainer of the experimental group will be an experienced facilitator who will conduct all training sessions (Y.L.). The training timetable will not include more than two 3-h sessions in one day. Physicians will have the opportunity to register in groups of three or individually. In the latter case, physicians will be assigned to groups according to geographical proximity.

#### CST sessions

The first session of the training program will include a general introduction to training and a modeling session. Sessions two to four will focus on appropriate communication skills for addressing uncertainty and hope according to a model detailed in a training manual. During sessions five to seven, participants will learn to transfer their newly-learned skills to clinical practice. Finally, during sessions eight to ten, skills learned during the training program will be consolidated.

#### CST program

The CST program will include theoretical information giving about uncertainty and hope in cancer care (based on psychodynamic, cognitive-behavioral and systemic theories), modeling and role-playing.

##### Theoretical information giving.

The CST trainer will provide theoretical information on communication skills needed to address uncertainty and hope in encounters with cancer patients. These skills will focus on assessing patient expectations about the future and restructuring patient understandings with appropriate information when needed; and assessing patient hopes about the future and supporting those which are realistic [47]. All skills will be based on a collaborative and bidirectional communication process between physicians and patients on topics such as disease prognosis or expected and unexpected medical, psychological and social effects of cancer treatments. A specific algorithmic theoretical model has been designed to aid physicians in the implementation of these communication skills.

##### Modeling.

During the first CST session, physicians will observe a 16-min video of a simulated interview in which the trainer acts as a physician communicating with a patient suffering from ﻿﻿an ﻿advanced cervical cancer. In the scenario, the patient has come for chemotherapy treatment and is requesting reassurance about treatment efficacy.

The modeling session will emphasize three factors: 1) physician attitudes necessary to address uncertainty and hope, 2) patient’s reactions to the discussion of uncertainty and hope and 3) the need to set up a safe and comfortable setting in which to model communication skills needed to address uncertainty and hope. After the video, physicians will be given one hour to debrief and react to the simulated interview.

##### Role-playing.

Throughout training, participants will be invited to participate in interactive role-playing with immediate and circular feed-backs [48]. Physicians will be asked to identify a clinical situation for the focus of the role-play situation. In session two to four, physicians will be asked to define a situation that would be highly uncomfortable in terms of uncertainty and hope management. In training sessions five to seven, physicians will be asked to identify clinical situations in which the transfer of learned communication skills would be difficult. Finally, in training sessions eight to ten, physicians will be asked to identify clinical situations during which the transfer of acquired skills would be uncomfortable.

During role-play, the physician who reports the clinical situation will take on the role of the patient. This will allow role-play to be as realistic as possible. The small group context will promote an interactive session. During role-play, the “patient” will be exposed to the ways that he and his two colleagues are communicating in repeated rotations. During each rotation, the facilitator will suggest alternative strategies that were taught in the theoretical model and shown in the modeling video.

##### Transferring to clinical practice.

Each training session will start with a 15-min summary of material learned since the beginning of the training program along with a debriefing from participants of attempts to transfer the learned skills to their clinical practice. Each training session will end with a 10-min summary of the skills learned during the session, the difficulties that may have been encountered, and a proposal for the transfer of newly learned skills to future encounters with patients.

### Assessment procedure

**Fig. 2:**
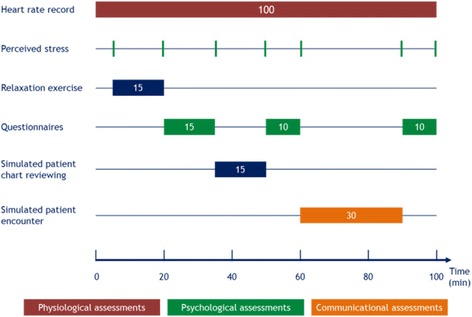
The seven steps of the assessment procedure: 1) continuous monitoring of heart rate, (2) relaxation exercise, (3) administration of questionnaires, (4) review of the simulated cancer patient medical chart, (5) administration of the second set of questionnaires, (6) encounter with the simulated cancer patient and (7) final set of questionnaires. Perceived stress will be measured seven times throughout the assessment procedure

The performance status, disease status and communication skills among cancer patients vary widely and as such, the use of standardized encounters with simulated patients has been recommended to assess the efficacy of CST programs designed for healthcare professionals [49]. The assessment procedure for the current study will involve the video recording of an encounter between the participating physician and a simulated advanced stage cancer patient. Participating physicians will be assessed individually. An investigator, not involved in the training program, will present each subject with questionnaires. The assessment procedure (Fig. 2) will include 7 steps: (1) continuous monitoring of heart rate, (2) relaxation exercise, (3) administration of questionnaires, (4) review of the simulated cancer patient medical chart, (5) administration of the second set of questionnaires, (6) encounter with the simulated cancer patient and (7) final set of questionnaires. Perceived stress will be measured seven times throughout the assessment procedure.

#### Simulated patient encounter

The simulated patient case was written by an oncologist and a psycho-oncologist at the medical oncology unit and the psycho-oncology clinic at the Jules Bordet Institute, Cancer Center of the Université Libre de Bruxelles. The simulated patient case was developed to increase physician uncertainty about predefined medico-psycho-social components and available evidence-based treatments. The simulated patient is a 36-year-old woman with advanced cancer. She is facing a third recurrence (hepatic metastasis) of a breast cancer that had previously been treated with surgery, hormone therapy, radiation therapy and chemotherapy. She has agreed to start a new chemotherapy treatment. The scenario specifies that the patient has requested a meeting with a physician to help her cope with her treatment decision. Participants will be instructed to address and respond to the concerns of the simulated patient and to take the time they need for doing that. The simulated patient will be played by an actress experienced in simulated patient encounters and will be trained to maintain a standardized script and behavior. Regular feedback sessions will be held to help the actress maintain reproducibility [50]. The simulated patient encounters will take place at the Communication Laboratory (LabComm) of the Centre de Psycho-Oncologie (Brussels, Belgium).

#### Psychological assessments

Participating physicians will be asked to complete a set of psychological questionnaires prior to reading the patient medical chart. Data on socioprofessional characteristics, practices in oncology and sense of mastery of the communication skills needed to address uncertainty and hope with cancer patients will be collected. A second set of psychological questionnaires administered immediately prior to the encounter with the simulated patient will gather information on the perceived realism of the medical chart of the simulated patient, agreement with the treatment decision, outcome expectancies on the medical, psychological and social status of the simulated patient, perceived uncertainty and hope regarding the medical, psychological and social outcomes of the simulated patient, and psychological reactions to uncertainty regarding the medical, psychological and social outcomes of the simulated patient. Finally, a third set of psychological questionnaires will be administered immediately after meeting with the simulated patient. These questions will assess to agreement with the treatment decision, satisfaction regarding the encounter with the simulated patient, and the sense of mastery regarding the communication skills used to address uncertainty and hope with the simulated patient. These psychological questionnaires will allow the assessment of predictors and correlates of communication skills learning used to address uncertainty and hope with the simulated patient.

#### Communication assessments

The encounter with the simulated patient will be video recorded and transcribed. Physician communication skills will be analyzed using three tools. The French communication content analysis software, LaComm (Centre de Psycho-Oncologie, Brussels, Belgium; http://www.lacomm.be/) analyzes verbal communication (in medicine in general and in oncology in particular) and identifies turns of speech and the type and content of speech. The explanation of how this software works has been detailed in previous publications [42, 51]. The Multidimensional analysis of Patient Outcome Predictions (MD.POP) is a reliable tool used to measure verbal expressions that address the clinical future of a patient during medical encounters. This coding system allows one to manually identify, code, and score detailed verbal content from a medical encounter transcript that addresses a patient’s clinical future. The detailed MD.POP codebook is available upon request. Finally, a specific interaction-process analysis system assessing communication skills addressing hope and uncertainty will be developed for the study [52].

#### Physiological assessments

Throughout all assessment procedures, physician heart rate will be monitored to assess the impact of the training program on the physiological arousal associated with communication about uncertainty and hope with the simulated patient. This assessment procedure has previously been used to measure the effect of CST on the physiological arousal of residents breaking bad news in a simulated task [53].

#### Statistical analyses

The primary outcome of the current study is the physicians’ increased communication performance after training during this encounter. A power analysis has been performed, based on a previous longitudinal study assessing physicians’ communication performance composite score in an encounter with a simulated advanced-stage cancer patient (Mean = 26; SD = 8) [54]. This power analysis was conducted considering 4 independent conditions according to the time (time 1 *versus* time 2) and the group (experimental *versus* control group). As there is no previous study assessing the efficacy of an intensive communication skills training program on physicians’ communication about uncertainty and hope, it was hypothesized that physicians in the control group will maintain a stable performance score from time 1 to time 2. It was also hypothesized that physicians in the experimental group will improve their performance score by 20% from time 1 to time 2. Sample size calculation has been based on an 80% power, a one-sided α = 0.05 t-test and an effect size of 0.65. Considering this power analysis, 60 evaluable physicians are therefore needed for the efficacy assessment. Considering a drop-out rate of 20%, 12 physicians should be moreover recruited (72 physicians in total). It should be recalled at this level that one trainer only will conduct the training of the experimental group. Secondary, to assess also the CST program efficacy, group-by-time effects will be performed using generalized estimating equation (GEE) on psychological, physiological and communicational assessments performed during the encounter with the simulated patient.

## Discussion

Due to medical progress, cancer is now recognized as a long-term chronic disease necessitating optimal communication between physicians and their patients to help patients cope with uncertainty and to promote hope regarding the future. However, due to a lack of specific training in medical curriculum [20], physicians frequently experience difficulties in communicating these issues with cancer patients.

The current paper describes a randomized controlled trial protocol assessing the efficacy of a CST program aimed at improving physician ability to communicate about uncertainty and hope in encounters with cancer patients. The CST program includes learner-centered, skills-focused, practice-oriented techniques with small groups of physicians (n = 3/group). The CST efficacy will be assessed at the communicational, psychological and physiological levels. Results from the study will provide information regarding CST techniques and content that will be beneficial in the development of programs to improve physician communication skills about uncertainty and hope with cancer patients.

The development of such CST programs will lead to positive outcomes for healthcare professionals, cancer patients and their relatives. Improving physician ability to communicate about uncertainty and hope with cancer patients may increase work satisfaction [30], decrease risk of staff burnout [25], improve cancer care delivery [32], limit risk of increased costs [25], limit use of healthcare services [31] and reduce healthcare professional decisional conflict and regret [8, 10–14]. Moreover, improving communication between physicians and patients about uncertainty and hope may increase patient satisfaction with healthcare and fulfill patient desire for information and maintenance of hope [6, 9, 34]. In addition, effective communication may improve patient adjustment to cancer [1, 7, 17], quality of life, maintenance of a positive outlook on future treatments and decrease decisional conflict and regret [1, 18]. Future studies should further assess the usefulness of the CST program used in the current study on all these outcomes.

## Abbreviations

CPO: Centre de Psycho-Oncologie
CST: Communication skills training
GEE: Generalized estimating equation
